# Supplementary figures and images for: Cathepsin Z is a conserved susceptibility factor underlying tuberculosis severity
Source: PLoS Biol. 2025 Sep 9;23(9):e3003377. doi: 10.1371/journal.pbio.3003377 (PMC12440229; doi:10.1371/journal.pbio.3003377)

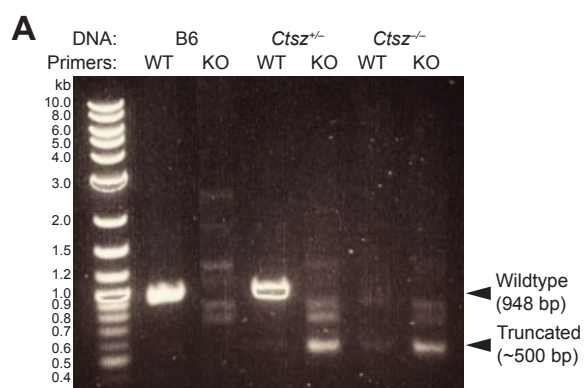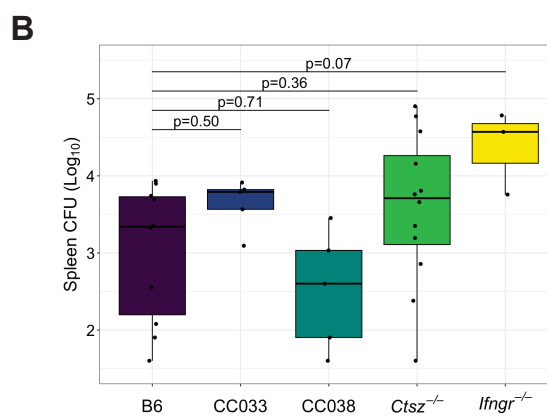

Supplement: S1 Fig — (A) Expression of wildtype and truncated Ctsz in tail sections from B6, Ctsz+/−, and Ctsz−/− mice. Approximate sizes of wildtype and truncated PCR products are indicated by black arrows. As previously described by Sevenich and colleagues, 2010 [91], exon 2 (containing the active site cysteine critical for the enzymatic activity of Ctsz), and a portion of intron 3 in Ctsz were deleted by homologous recombination and substituted by a cassette comprising an independent ribosomal entry sequence (IRES). External confirmation of these results was obtained by probing the lacZ reporter gene present in the inserted IRES vector. (B) Bacterial burden measured by dilution plating from spleen homogenate 4 weeks after aerosol infection with Mtb H37Rv (n = 3–12 per strain; all males except B6 and Ctsz−/− groups, which included both sexes in equal proportion). Hypothesis testing was performed by one-way ANOVA and Dunnett’s post hoc test on log10-transformed values. The data underlying this figure can be found in S1 Data sheet S1B. (PDF) [file pbio.3003377.s001.pdf]

**A**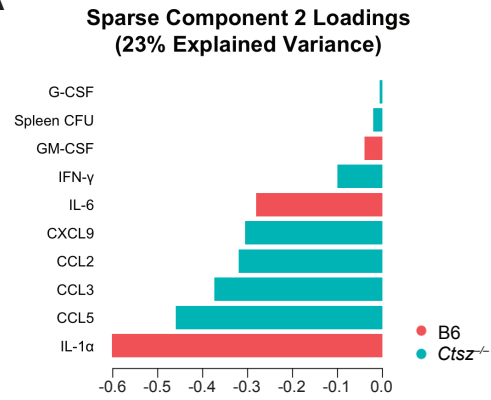**B**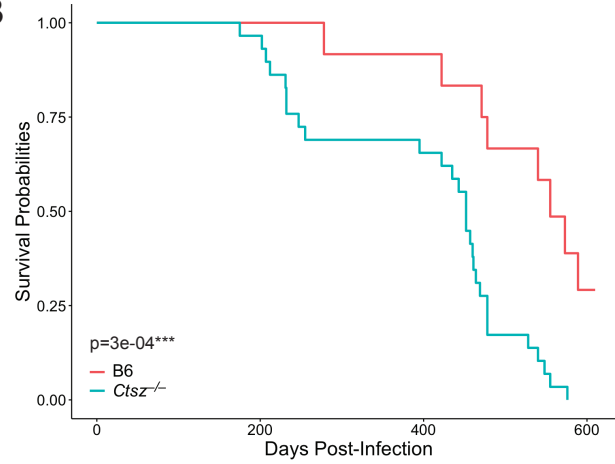**C**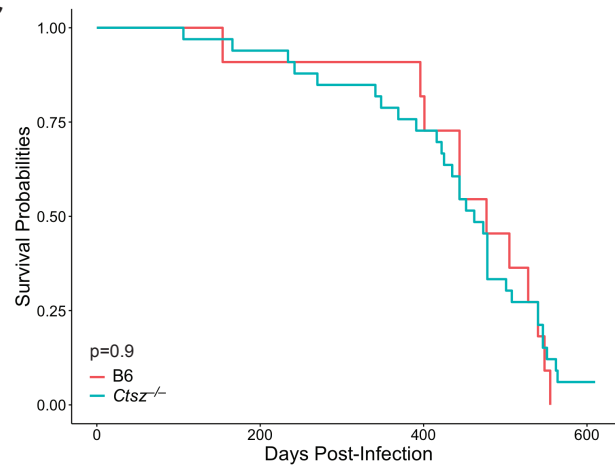

Supplement: S2 Fig — (A) Phenotype loadings contributing to sparse component 2. Mice were sacrificed at 2, 3, 4, and 8 weeks after aerosolized Mtb infection. Data are from two experiments with n = 6–14 mice per genotype, representative of both sexes, at each time point. Kaplan–Meier survival estimates of aerosol-infected B6 (n = 23) and Ctsz−/− mice (n = 62) across two independent experiments, among (B) male and (C) female mice. Hypothesis testing was performed using a log-rank test. Equal proportions of both sexes were included. The data underlying this figure can be found in S1 Data sheets 2ABCDEF_S2A and 2G_S2BC. (PDF) [file pbio.3003377.s002.pdf]

**A**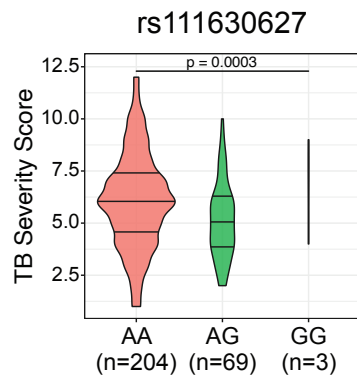**B**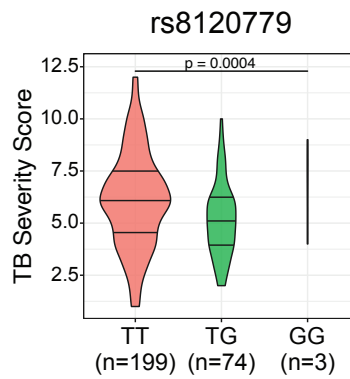**C**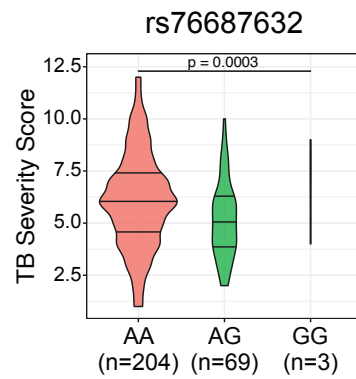**D**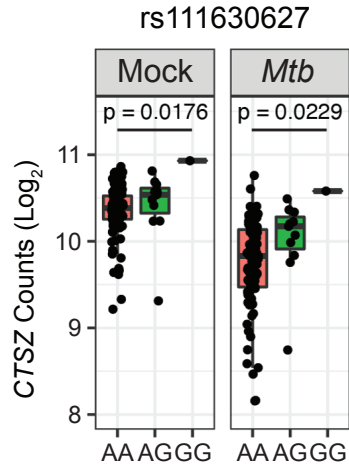**E**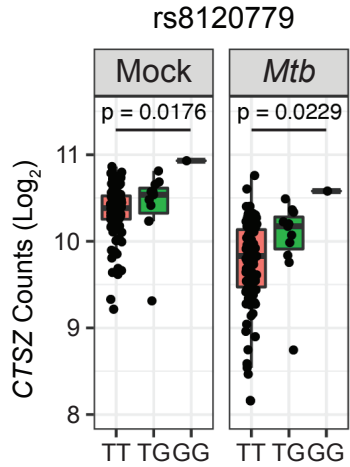**F**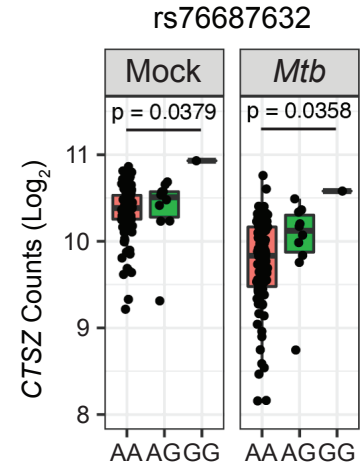

Supplement: S3 Fig — Comparison of TB severity, measured using Bandim TBScore, by genotype for (A) rs111630627, (B) rs8120779, and (C) rs76687632 SNPs. Expression of each allele of each SNP was assessed by RNA-Seq at 6 h after mock and Mtb infection in human-derived monocytes. CTSZ expression by monocytes harboring the minor allele for each SNP was significantly increased following both infection conditions for the (D) rs111630627, (E) rs8120779, and (F) rs76687632 SNPs. eQTL effects were assessed with a linear mixed effect model in kimma to account for sex, age, RNA-Seq batch, genotypic principal components 1 and 2, and kinship. The data underlying this figure can be found in S1 Data sheet 4CD_S3DEF. (PDF) [file pbio.3003377.s003.pdf]

A

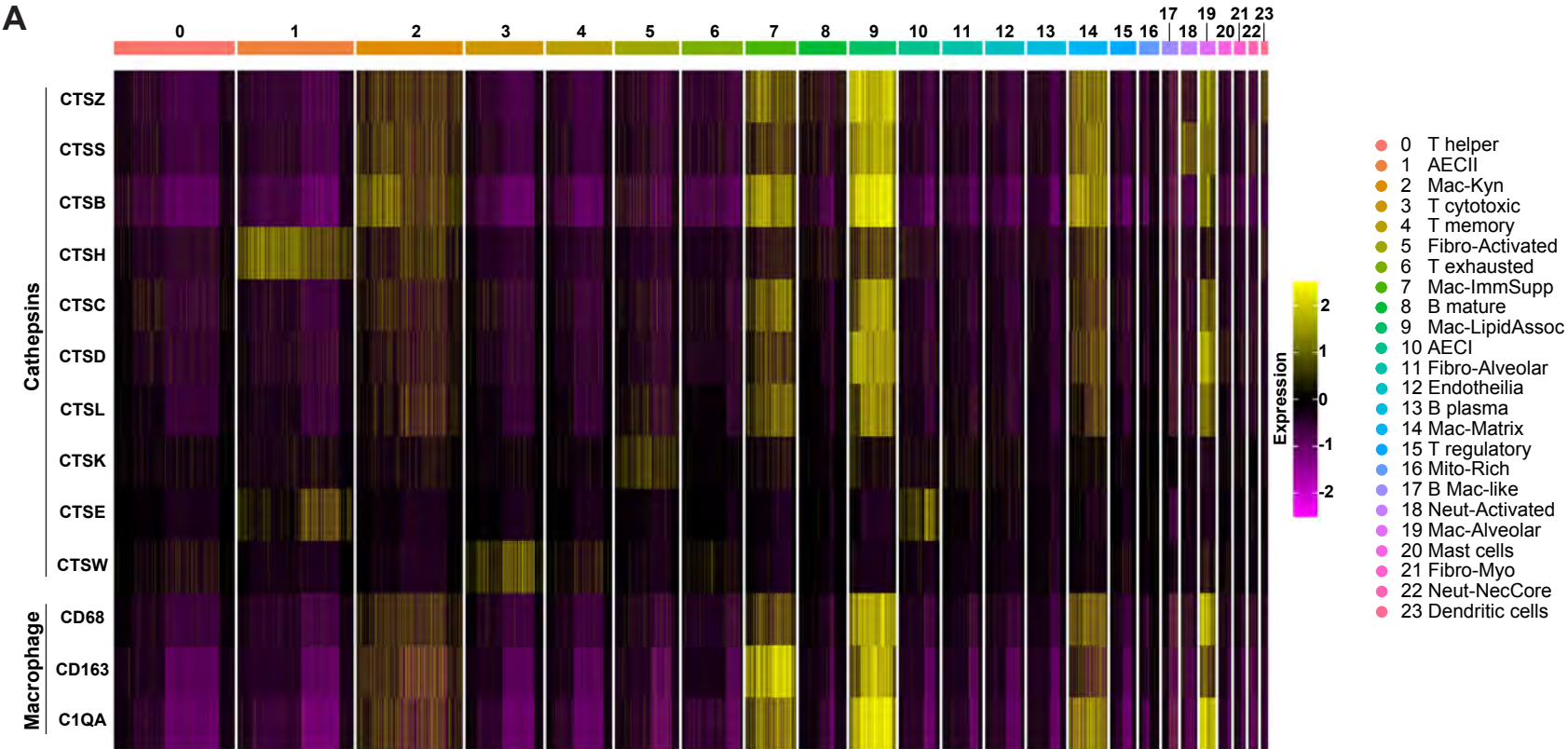

B

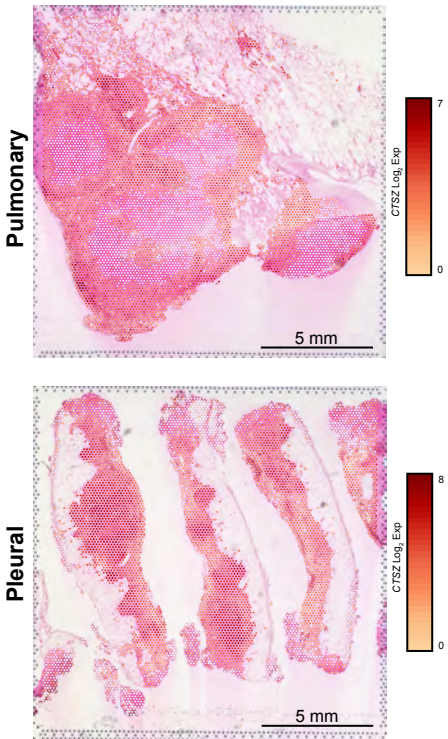

Supplement: S4 Fig — (A) Heatmap depicting mRNA expression levels of several cathepsins and macrophage markers across unsupervised scRNA-Seq cell clusters. (B) The positional distribution of CTSZ expression in human Mtb granulomas as determined by Visium v2 spatial mRNA-Seq of Eosin-stained biopsy tissue sections from two patients with TB. This figure was generated by re-analysis of previously published data from Pyle and colleagues, 2025 [65]. Cell clusters were annotated by the authors. Data from Pyle and colleagues, 2025 are available in the NCBI GEO under accession numbers GSE296399 and GSE296400. (PDF) [file pbio.3003377.s004.pdf]

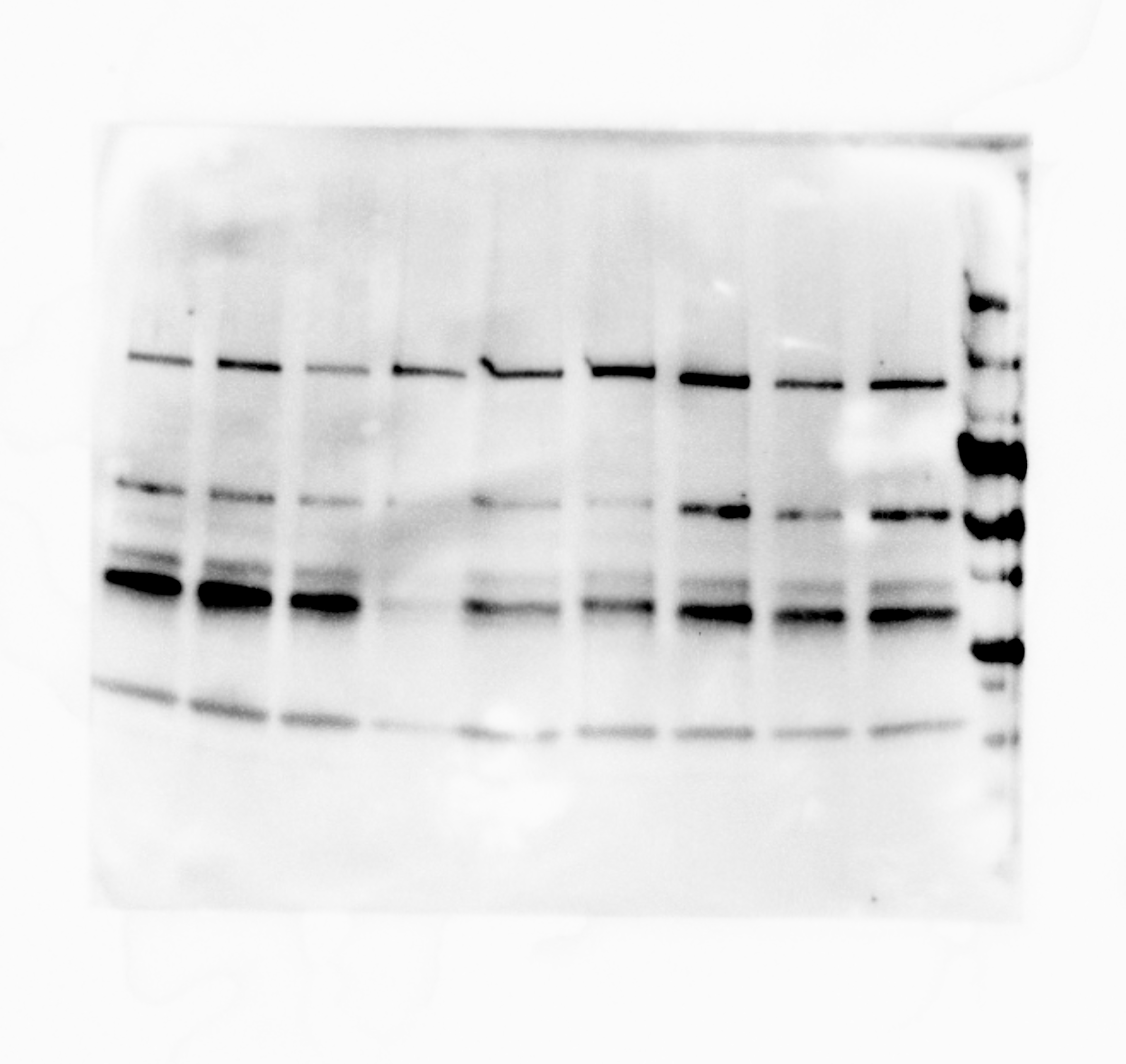

Supplement: S1 File — (TIF) [file pbio.3003377.s005.tif]

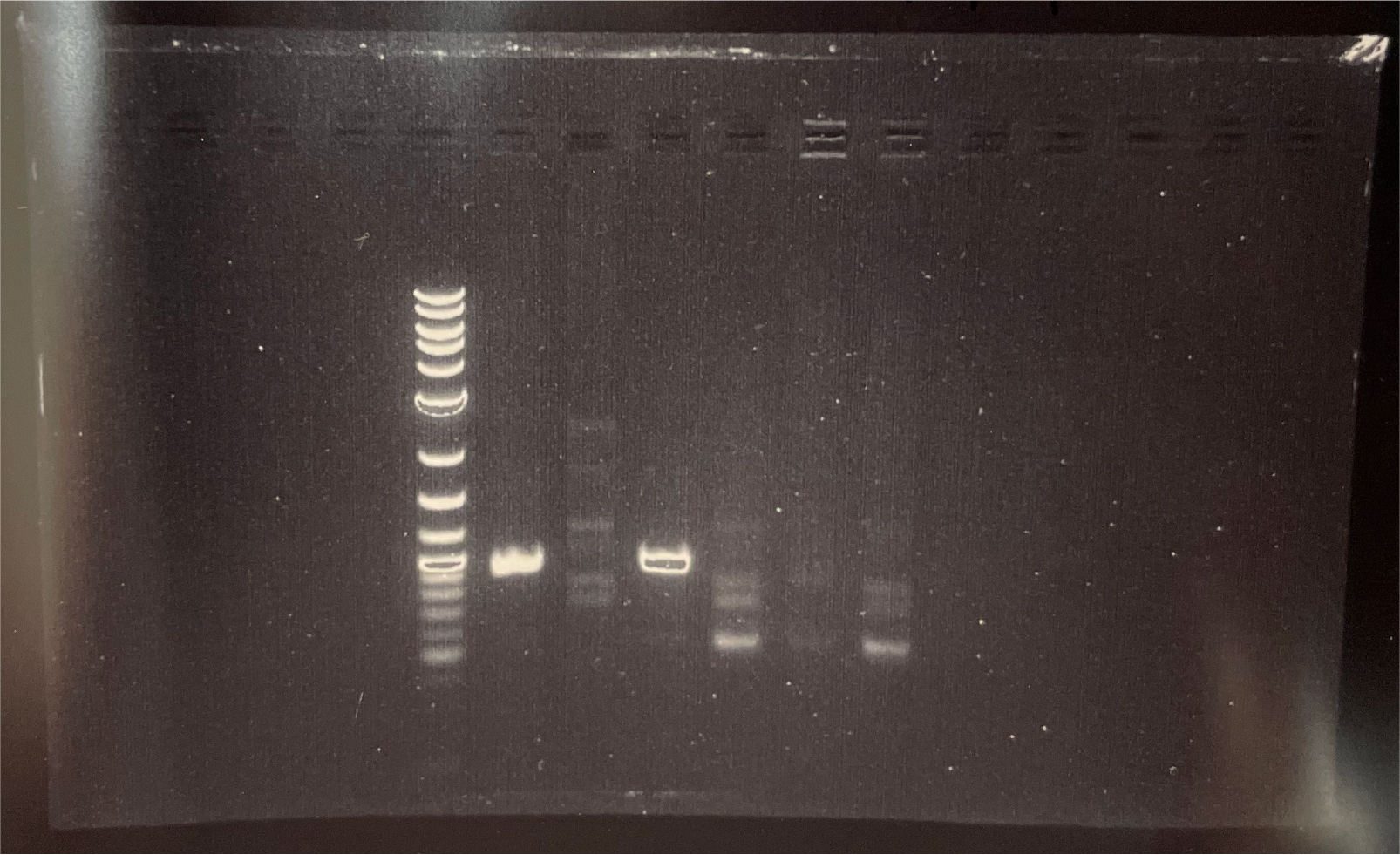

Supplement: S2 File — (PNG) [file pbio.3003377.s006.png]

Fig 1B

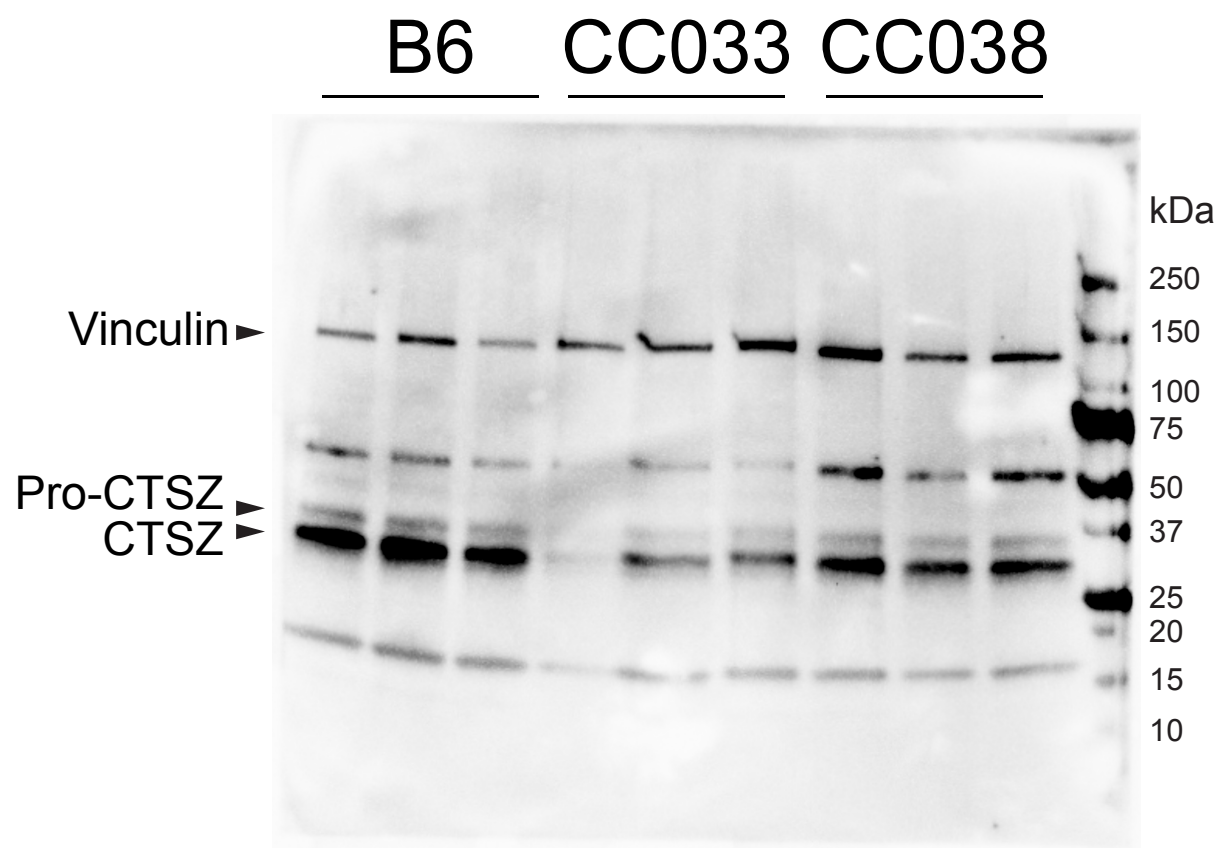

S1A Fig

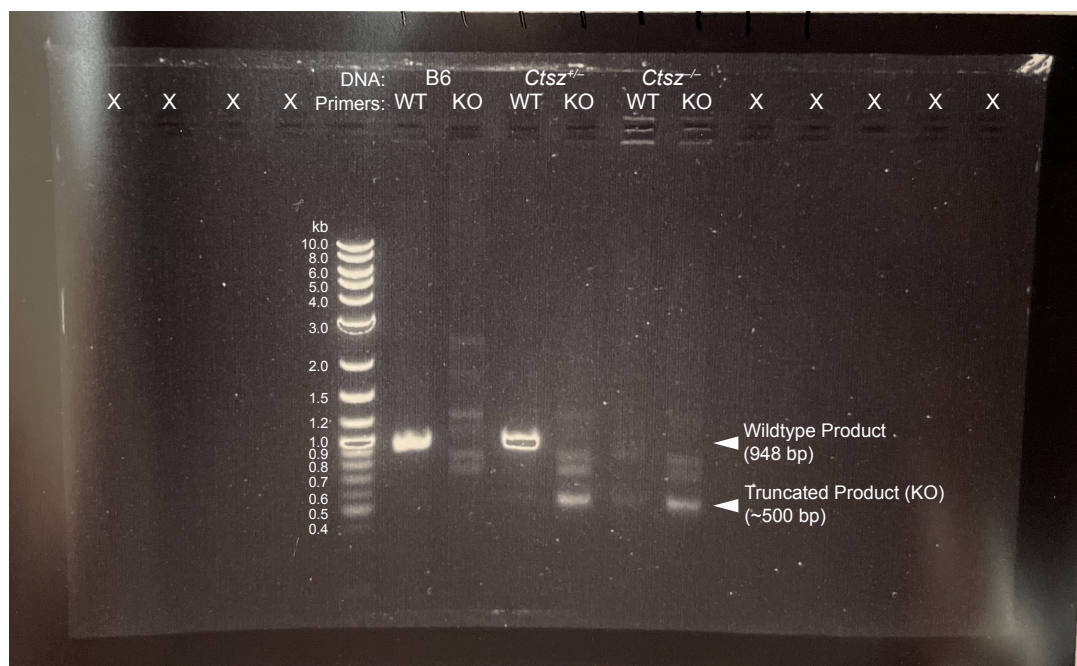

Supplement: S1 Raw images — Annotated raw images for Fig 1B and S1A Fig. (PDF) [file pbio.3003377.s008.pdf]
