# Supplementary material for: Cathepsin Z is a conserved susceptibility factor underlying tuberculosis severity
Source: PLoS Biol. 2025 Sep 9;23(9):e3003377. doi: 10.1371/journal.pbio.3003377 (PMC12440229; doi:10.1371/journal.pbio.3003377)
Supplement: Attachment — Submitted filename: 2025.07.21_Response to Reviewers.docx [file pbio.3003377.s012.docx]

**RESPONSE TO REVIEWERS:**

We thank the editor and reviewers for their thoughtful and constructive feedback. As noted by the editor, “*the majority of reviewers are positive about the relevance and novelty of the study*”. Reviewer #1 described the manuscript as providing “*important, novel, and mostly convincing information.*” Reviewer #2 acknowledged the “*The merit of this article is to establish the functional impact of genetic variations (null alleles or SNPs) in the Ctsz gene and the susceptibility to TB, and to link it to CXCL1 expression*.” Reviewer #3, along with Reviewer #2, emphasized the strengths of our approach, highlighting its “*basis in sophisticated and rigorous mouse genetic analysis, the leveraging of multiple public datasets to analyze their candidate gene, and integration of new clinical data*” and noting “*article is well-written and structured, especially in regard to the multiple sources and nature of the data*”, respectively. Furthermore, Reviewer #2 recognized the broad impact of our findings, stating that “*the findings will be interesting to researchers in the TB field and beyond considering the implication of Ctsz in multiple infectious, immune, neurodegenerative or cancer diseases*”, a perspective that underscores our study’s cross-disciplinary importance and reinforces its suitability for publication in *PLOS Biology*.

We have addressed each of the three reviewers’ concerns, both technical and conceptual. Our revisions include a new analysis of western blots from the CC mice in which we delineate relative expression of each cathepsin Z isoform, showing that both the pro-CTSZ and mature CTSZ isoforms are compromised in the CC mice carrying the NOD genotype at this locus. This analysis reframes the impact of the study and its findings and highlights the unique translational links from mouse genetic and functional studies to human genetic variation and disease susceptibility.

One of the three reviewers felt that the initially submitted version of the manuscript did “not quite generate the level of excitement” required for a general audience. They nonetheless appreciated the “sophisticated and rigorous mouse genetic analysis, the leveraging of multiple public datasets to analyze their candidate gene, and integration of new clinical data.” We feel that the innovative use of mouse systems genetics and the Collaborative Cross, pinpointing a specific locus and a subsequent candidate gene underlying differences in TB disease severity, as well as the finding that genetic variation at this locus in mice and humans influences TB severity, likely through induction of a chemokine previously associated with disease severity, will be exciting to a diverse range of individuals across the scientific community. To name a few, geneticists studying infectious disease in mouse models and other model organisms, immunologists exploring the biology underlying immune mechanisms of homeostasis and host protection, human geneticists and global health specialists studying genetic variation in populations bearing high infectious disease burden, as well as infectious disease scientists and clinicians. Since *M. tuberculosis* remains the single deadliest human pathogen worldwide, we believe that the findings will be particularly interesting to general readership.

**To strengthen the connection to human disease, we now include newly generated single-cell and spatial transcriptional data and analysis from additional human *Mtb* granulomas.** Consistent with the initial submission in which we performed antibody staining on one of these human specimens, we now use a combination of single-cell RNA-sequencing of human granulomas and spatial transcriptional profiling to highlight the positioning of *CTSZ* induction within the granuloma microenvironment. Across four independent human TB biopsy specimens, we find that human *CTSZ* is consistently expressed in granuloma-associated macrophages and particularly enriched in a subpopulation of lipid-associated macrophages located adjacent to the necrotic core. We include entirely new panels with these results and analyses, which, if accepted and published, would represent one of the first published paired single-cell and spatial transcriptional analyses from human TB lung granuloma samples (although there are a couple preprints from earlier this year, and this is within the context of a larger, more comprehensive analysis that will be published separately).

We have also rewritten the text and its framing to highlight key aspects noted by the reviewers, including further discussion of sex differences in TB, a well-known epidemiological aspect of TB that is recapitulated here in the animal model, and have sought to clearly articulate the anticipated impact of this work.

**REVIEWER #1**

Various association studies and some in vitro work have been suggesting an involvement of cathepsin Z (CTSZ) in the susceptibility for mycobacterial infection causing tuberculosis (TB). Based on their previous work on recombinant inbred mouse strains, which identified a TB susceptibility locus on mouse chromosome 2, the authors of this manuscript now identify and validate CTSZ as important mediator of this susceptibility in vivo. This is further connected to the inflammatory response of macrophages, especially increased CXCL1 upon CTSZ deficiency. Finally, the paper provides evidence for variants in human CTSZ associated with TB severity in patients. Overall the manuscript provides important, novel, and mostly convincing information.

We thank Reviewer #1 for their thoughtful and constructive feedback. In response, we now separately quantify the pro- and mature CTSZ isoforms present in our Western blots in the new panels Figure 1C & D. We also removed potentially confusing supplemental data, which differed from the Figure 1B Western blot in sample type and preparation.

We have expanded the Discussion to address the observed sex-specific survival differences, incorporating relevant literature on observed sex disparities in human TB and murine bone marrow-derived macrophage (BMDMs).

We have also revised the Methods to clarify the *Ctsz^–/–^* mouse line nomenclature and genetic background and have provided previous evidence from our work supporting our hypothesis that 129-derived passenger sequence is unlikely to confound our results. Lastly, we have improved the clarity of genotype results presented in Figure S1A by labeling PCR products and updating the legend.

We appreciate Reviewer #1’s input, which has strengthened both the clarity and rigor of our manuscript.

Major issues:

1)	CTSZ Western blots (Figure 1B/C; Sup. Figure 1B). Background: Cathepsin Z is produced as a pre-pro-protein. After cleaving of the signal peptide in the ER, Western blots usually detect the inactive Pro-CTSZ and the active mature CTSZ. The mature enzyme is generated by cleaving of a 60 amino acid "activation peptide" from the Pro-CTSZ. This is causing a band shift of 4-5 kDa. Some cell types with high CTSZ activity may predominantly (or only) show the active enzyme. 
Questions: The Western blots show multiple bands - what bands correspond to which form of CTSZ? What bands were used for quantification shown in Fig 1C? Supp. Figure 1B (intended to proof CTSZ ko) lacks loading controls and shows somewhat different band pattern to Fig 1B. 
Recommendation: For detection of mouse CTSZ in mice use R&D AF 1033; the "all-purpose" AF934 is inferior when used on mouse tissue samples.

We thank Reviewer #1 for this detail. The top band corresponds to pro-CTSZ, and the lower band corresponds to mature, active CTSZ (PMID: 17065156). After quantifying both the pro-form and active form of CTSZ separately from Figure 1B, we still find CC033 and CC038 have significantly lower levels of both forms of CTSZ than B6 by Dunnett’s *post hoc* test after adjustment for total protein level against vinculin loading controls. Within Figure 1, we now include plots quantifying the pro-CTSZ and mature CTSZ by host genotype as Figures 1C and 1D, respectively.

To mitigate confusion related to distinct CTSZ appearance via Western blot using distinct sample types and preparations (whole tissue vs. BMDM lysate), we have also removed the Western blot originally submitted as Figure S1B for the resubmission. The PCR depicted in Figure S1A along with prior evidence (PMID: 20133781) provide sufficient support of *Ctsz* ablation in the *Ctsz^–/–^* mouse line.

2)	It is striking that the reduced survival in experimental TB is only evident for male CTSZ ko, but not for females of this genotype. Figure 3E+F, analyze macrophages of male mice for CXCL1 production. Is there a CXCL1-difference in female-derived wt and CTSZ ko macrophages? Whatever the outcome it could serve as a starting point to discuss this important sex-dependent outcome (the discussion of this point is not sufficiently elaborated in the current manuscript).

We thank Reviewer #1 for bringing up the important observation of sex-specific differences in infection outcomes. It is well documented that male mice are more susceptible to infection with *Mtb* (PMID: 28887521, 32198367) and that men bear a greater portion of the global TB burden than women (PMID: 27598345). Sex differences are increasingly recognized as a critical factor underlying TB pathogenesis and host immune responses (PMID: 35842266).

It has also been shown that BMDMs derived from male mice exhibit enhanced pro-inflammatory response following stimulation with LPS compared to BMDMs derived from female mice (PMID: 34867999), yielding higher expression levels of several pro-inflammatory cytokines, including TNF-α and IL-1β. Although authors of the previous study did not directly assay CXCL1, the chemokine has been shown to contribute to active IL-1β levels in murine BMDMs following LPS stimulation (PMID: 28739876). In a preliminary *Mtb* infection experiment in age-matched female siblings, we observed that the *Ctsz^–/–^* BMDMs produced less CXCL1 than BMDMs derived from the matched *Ctsz^+/+^* sibling. However, as these results reflect only one preliminary experiment, we cannot conclusively state whether there is a significant sex difference in macrophages at this time. We agree that the sex-stratified survival data presented in the manuscript serve as a starting point to discuss sex-specific outcomes. These data provide a basis for future mechanistic studies to dissect precisely how *Ctsz* influences immune responses in a sex-dependent manner and underscore the need for stratifying preclinical infection studies by sex to fully elucidate host-pathogen interactions in TB. We have now elaborated on the need for further investigation into the biological underpinnings of the sexual dimorphism observed in *Ctsz^–/–^* mortality risk in the Discussion section of the revised manuscript.

Minor issues:

1)	Ctsz-/- Mice: Please give the appropriate full nomenclature of the mice according to the mouse genome informatics database in the method section (as for the IFNg mice). Further note that mice had been generated on a mixed genetic background and were backcrossed to C57BL/6. This has been first reported in PMID: 25274726.

We thank Reviewer #1 for pointing out the lack of clarity in this section. Unfortunately, we could not locate a mouse genome informatics (MGI) entry for the backcrossed B6 line harboring the targeted *Ctsz* mutation (see MGI:4436876, the entry for the mutation itself). As our group received the *Ctsz^–/–^* line from Yates Lab and to avoid introducing incorrect information on the generation of the line, we have added additional language to our Materials & Methods section to clarify that the generation of the line is described in Sevenich et al., 2010 (PMID: 20133781), aligning the approach taken by Yates Lab in discussing the generation of this line in two previous publications (PMID: 28486971, 34864055).

2)	Please consider/discuss: The mice have been generated in HM1 ES cells (129P2/OlaHsd background). The mice were carefully backcrossed; but the original background is likely to be maintained around the CTSZ knock-out locus (for which the genotyping selects). How would this affect the results?

We thank Reviewer #1 for this insightful question. We cannot definitively articulate the impact of the surrounding 129P2/OlaHsd sequence on TB outcomes *in vivo* without functional dissection of this locus and subsequent infection experiments. Nevertheless, we have reported evidence suggesting that genomic sequence inheritance in the *Tip5* region (which contains *Ctsz*) from a closely related 129 line has minimal impact on *Mtb* susceptibility *in vivo*.

129S1/SvlmJ, a close phylogenetic relative of 129P2/OlaHsd by whole-genome sequencing analysis (PMID: 36163035), is one of the founders of the genetically diverse Collaborative Cross (CC) panel. In our previous CC infection screen that uncovered the *Tip5* QTL, we used Best Linear Unbiased Predictor (BLUP) analysis to show that genomic inheritance from NOD/ShiLtJ mice across the *Tip5* region promotes susceptibility to *Mtb* infection (PMID: 35112666, see Figure 2E). Of note, inheritance of genomic sequence from 129S1/SvlmJ across the *Tip5* region did not contribute to the *Tip5*-associated susceptibility phenotype that we later linked to *Ctsz*. This evidence suggests that genomic contributions from 129P2/OlaHsd across the broader *Tip5* region (encompassing passenger sequence likely harbored by the *Ctsz^–/–^* line) would be more likely to reduce the susceptibility phenotype than to enhance it in the *Ctsz^–/–^* line, provided that neither the 129P2/OlaHsd line in general nor the isolated portion of 129P2/OlaHsd within the region surrounding the targeted *Ctsz* mutation have developed private mutations in comparison to 129S1/SvlmJ that modify clinical outcomes of TB.

3)	Supplement. Figure 1A: Please label the PCR bands (what are the specific PCR products?) Please explain in the figure legend to a general reader, why the PCR products form (or not form) in the WT and KO PCRs.

We thank Reviewer #1 for pointing out this lack of clarity. We have updated the Figure S1A panel and expanded the legend to highlight wildtype vs. truncated PCR products and their differences.

------------------------------------

**REVIEWER #2**

This study by Maede et al. has investigated the impact of Cathepsin Z loss of function on the susceptibility to M. tuberculosis. Their focus on Ctsz comes from previous QTL mapping on Collaborative Cross mice pointing at a small region of chromosome 2 and from converging evidence from human GWAS linking SNPs in Ctsz and susceptibility to TB. This study assembles experimental data produced by the authors mostly on mice but also on human samples and data from previously published study in humans, macaques and zebrafish. They identify CXCL1 overexpression in monocytes/macrophages as a prominent perturbation in Ctsz KO mice. In a human cohort, they show association between TB severity and Ctsz expression in patient-derived monocytes. Finally, they show that Ctsz is expressed in human granuloma macrophages, at the interface between the host and the pathogen.
The merit of this article is to establish the functional impact of genetic variations (null alleles or SNPs) in the Ctsz gene and the susceptibility to TB, and to link it to CXCL1 expression. While the association between severe TB and Ctsz variants has been reported in several studies, experimental evidence of their role was missing.

The results are well presented (with some improvements suggested below) and the conclusions are supported by the data. The article is well-written and structured, especially in regard to the multiple sources and nature of the data. The findings will be interesting to researchers in the TB field and beyond considering the implication of Ctsz in multiple infectious, immune, neurodegenerative or cancer diseases.

We thank Reviewer #2 for their thoughtful and encouraging review. We appreciate the recognition of our contribution to establishing a functional role for *Ctsz* in TB susceptibility and CXCL1 regulation, and for highlighting the strength of our multi-species, multi-modal approach.

In response to Reviewer #2’s comments, we have expanded the Discussion to more precisely articulate mechanisms we suspect may constitute the “CTSZ–CXCL1 axis,” potentially explaining the relationship between early inflammation and later mortality *in vivo*. We also acknowledge the potential contribution of other genetic loci in CC033 and CC038 mice to TB susceptibility, an inherent challenge of using the genetically heterogeneous CC panel.

Comments from Reviewer #2 identified several points for clarification throughout the manuscript, including gene nomenclature, ortholog relationships, pathogen-specific plot labels, statistical annotations, and breeding strategy. We have updated the manuscript to reflect these changes. Moreover, we have revised language around the role of *Ctsz* to reflect its function as a determinant, as suggested.

We are grateful for Reviewer #2’s insight and careful review of our work, which has removed several potential points of confusion for future readers.

Main comments:

Although this is not the main focus of the paper, it is interesting to note (Fig 1D) that lung bacteria load is much higher in CC033 and CC038 than in Ctsz -/- mice, suggesting that they carry other susceptibility genes.

We agree with this observation. Several TB-relevant loci have been identified in the CC panel (PMID: 35112666) and the sister Diversity Outbred (DO) panel (PMID: 38861581). It is entirely possible that CC033 and CC038 may harbor one or more additional loci that could enhance *Mtb* susceptibility compared to *Ctsz^–/–^* mice on a primarily C57BL/6J background. One of the canonical challenges of working with these genetically diverse mice is that QTL mapping of polygenic or epistatic phenotypes, like bacterial burden and weight loss, can yield strong mapping of one or two genomic loci or weak mapping of several relevant loci. Polygenicity and epistasis in QTL mapping studies continue to pose significant challenges to yeast geneticists (PMID: 35147078) and human geneticists alike (PMID: 34475573). For these reasons, we prioritize QTL for investigation from our previous infection screens by leveraging inter-study comparisons to identify highly immunomodulatory regions that are found to repeatedly impact TB outcomes *in vivo* (PMID: 39034171, see Figure 2). Through this approach, and by leveraging other published datasets, as in the present manuscript, we aim to draw evidential support from the work of other groups to overcome the noise inherent to the genetic mosaicism of the CC/DO mice. There remain several outstanding QTL for future investigation from our initial CC infection screen, including additional loci that may be associated with variation in burden outcomes between CC033 and CC038.

The authors mention a "CTSZ-CXCL1 axis" but do not establish a functional link between the two genes, despite the observation that CXCL1 expression is higher in Ctsz-deficient mice. What is the basis for this "axis"? What could be the mechanisms (direct or indirect?) linking these two genes and the potential downstream consequences? Expanding the discussion on this aspect of the work would point at future directions of research.

We thank Reviewer #2 for this critique and have expanded our Discussion section with our hypothesis for a possible mechanism linking CTSZ activity with CXCL1 production.

Ctsz deficiency results in increased lung bacterial burden and CXCL1 expression in the early phase of infection (W2-W4, Fig 2A, 2F) but does no longer at W8 (at least not significantly). However, it results in increased mortality rate starting 200 days p.i. (Fig 2F), as stated in lines 251-253, and 373-375. Could the authors discuss this observation and suggest mechanisms? Do they have data on bacterial burden at later time points that would support chronically (though modestly) elevated bacterial burden? Or could it be due to unfavorable events in the early phase of infection that determine the final outcome?

We thank Reviewer #2 for this thoughtful comment. While we observed significantly elevated bacterial burden and increased CXCL1 expression in Ctsz-deficient mice at timepoints throughout the early phase of infection (W2, W3, W4), this bacterial burden difference was no longer statistically significant at W8. In contrast to earlier timepoints, the W8 comparison was statistically underpowered due to smaller sample size (n=6 *Ctsz^–/–^* mice). Therefore, despite the higher median CFU values among *Ctsz^–/–^* mice, a significant difference between genotypes could not be detected at W8.

Importantly, we do not routinely collect lung CFU data at terminal timepoints for survival cohorts, as mice are typically euthanized based on humane endpoints and not at standardized timepoints with matched controls. However, previous studies have shown that *Mtb*-infected mice often exhibit high bacterial burden at the time of death (PMID: 38861581), suggesting that the increased risk of mortality observed in *Ctsz^–/–^* mice likely coincides with greater levels of bacterial burden at these terminal endpoints despite the less substantial difference observed at the 8-week timepoint.

Taken together, we hypothesize that early inflammatory dysregulation, signified by elevated CXCL1 levels, sets the stage for poor disease control and worsened clinical outcomes, ultimately contributing to increased mortality risk. This model is consistent with the idea that early immune responses can have lasting impacts on disease trajectory and severity, even if bacterial burden converges at mid-stage infection (PMID: 21249199, 27763255).

Minor comments

Lines 86-88: the sentence mixes two distinct points. First, inbred mice (including CC) are genetically identical within strains, which allows replication, investigating sex differences and disease incidence. Second, common laboratory strains were developed from a small pool of progenitors and therefore sample a small amount of genetic polymorphism, much smaller than that of the human population.
We thank Reviewer #2 for this critique and have split the sentence into two separate sentences to clarify both points made by the reviewer above.

Line 100: We therefore sought to determine WHICH genes found

This sentence has been updated accordingly.

Line 127: Are cathepsin X or P identical to cathepsin Z (confusing nomenclature) or different members of a family?
We agree that the nomenclature is confusing. To our knowledge, these names for cathepsin Z are used interchangeably, with some of the most significant early work articulating the structure and immune functions of the protein referring to it as cathepsin X (PMID: 9738465,19262176). The manuscript text has been updated for clarity.

Line 131: is rhesus monkey ZNF831 homologous to mouse Zfp831? Please explain.

We thank reviewer #2 for this query. The cited study, Ahmed et al., 2020 (PMID: 31996462), identifies *ZNF831* as a high confidence human and macaque orthologue of mouse *Zfp831* using Ensembl-based orthologous gene matching approaches. A note that *ZNF831* is orthologous to mouse *Zfp831* has been added to the manuscript.

Lines 213-214: specify that uninfected mice are denoted as W0

This note has been added to a sentence in the Figure 2 legend that describes the uninfected mice in Figure 2C in greater detail.

Line 242, Fig 2C: the heatmap does not show obvious differences between B6 and Ctsz-/- mice. This data should be added as a plot to Fig 2F.

We would contend that Figure 2C shows that *Ctsz^–/–^* mice have elevated inflammation at week 4 and 8 post-infection compared to B6, which may (as reviewer #3 pointed out) be driven by bacterial burden. We would also like to highlight that the same data depicted in Figure 2C, namely the rows labeled “Lung CFU”, “Spleen CFU”, and “CXCL1”, are currently also depicted in Figures 2A, 2B, and 2F, respectively.

Mine 279, Fig 3E: add the type of infection as a plot label. log-tranform data as in Fig 2F to better visualize low levels.

In the revised manuscript, the pathogen used for Figures 3E and 3F has been added as plot labels, and plotted values have been log-transformed.

Line 311 and Fig4B, C, D: show statistical comparison on the plots. In the text, specify that the effect of the T minor allele is based on the comparison between the CC and CT groups.

These p-values have been added to the plots, and additional clarity has been added into the text.

Lines 319-321: Together, these data suggest that THESE CTSZ variants are associated with both TB disease severity.

This sentence has been updated accordingly.

Lines 375-376: Explicitly describe the effect. "We show" is exaggerated by comparison with lines 297-298: "suggest an interaction"

We thank Reviewer #2 for this point for clarification. The direction of effect is now explicitly stated in the sentence at issue.

Lines 412-414: Do the authors consider Ctsz just as a "correlate"? From the data, they could argue it is a determinant.

The authors appreciate Reviewer #2’s confidence based on the evidence presented. The authors aim to interpret results conservatively, but we agree that the term “determinant” is appropriate to the data presented. The sentence has been adjusted to use the term “determinant.”

Lines 463-466: by crossing heterozygotes to keep the two alleles in the same colony?

The authors thank Reviewer #2 for critical review of the Methods. Additional text has been added to clarify the breeding scheme used to generate the mice used for *ex vivo* macrophage infections.

------------------------------------

**REVIEWER #3:**

The work of Meade et al is an elegant use of mouse genetics to identify the host determinants of susceptibility and resistance to M. tuberculosis (Mtb).  In their prior work they had leveraged the genetic diversity of collaborative cross (CC) mice to identify an Mtb susceptibility locus (Tip5). Here they report that one of the genes, cathepsin z (Ctsz), acts to restrict Mtb replication in mice, and they perform analysis on human genetic data that also finds an association of CTSZ with host defense. This study has several strengths, including its basis in sophisticated and rigorous mouse genetic analysis, the leveraging of multiple public datasets to analyze their candidate gene, and integration of new clinical data. However, there are also some weaknesses in this manuscript. The mechanistic details are somewhat sparse, even for a Short Report manuscript. In addition, while the work does make significant advances, it  does not quite generate the level of excitement that would appeal to a broad audience needed for publication in a very high-impact journal like PLOS Biology.

There were multiple strengths to this study including a systematic use of public data to narrow candidates (Fig 1A), carefully controlled animal experiments (Fig 1,2), analysis correlating human SNPs to expression of CATZ. Review concerns are listed below.

We thank Reviewer #3 for their thoughtful and constructive review. We appreciate the recognition of the study’s strengths, including both classical and next-generation mouse models, integration of published datasets, and incorporation of novel human clinical data.

In response to the reviewer’s main concern regarding novelty and general interest, we clarified how this work builds upon prior studies by reporting the first functional validation of *Ctsz* as a TB susceptibility factor *in vivo*. In addition to the antibody staining of CTSZ in human *Mtb* granulomas, we now provide new insights into *CTSZ* induction from recently published transcriptomic analyses with single-cell and spatial resolution, revealing clear *CTSZ* induction at the periphery of the necrotic core. We maintain that this work is a fulfillment of the original aim of the genetically diverse CC panel, bringing discovery from a genetic association to translational mechanistic understanding. We also highlight the importance of CTSZ for several other endogenous and infectious diseases and the broad relevance of genetic diversity and sexual dimorphism to a general biology audience.

To address mechanistic questions, we provided further information on *Ctsz* SNVs in mice, clarified limitations in directly linking *Ctsz* to bacterial burden in BMDMs, and expanded discussion of CXCL1 dysregulation as a key downstream signature of *Ctsz* disruption.

We addressed all minor comments by clarifying figure content, improving statistical annotations, sharing information on heatmap scaling, and adding new spatially resolved scRNA-seq data from human *Mtb* granulomas in revised figures (e.g., Figure 4E-H, Figure S4) to bridge RNA and protein-level findings in patients with TB.

We are grateful for Reviewer #3’s feedback, which has materially enhanced the results presented in this manuscript and encouraged thoughtful discourse on the impact of our work.

Major Issues

*	While identifying role for Ctsz in vivo an advance for TB researchers, probably not high-impact enough for a general audience. Impact is also somewhat decreased by the existing (though admittedly not very in-depth) work done on Ctsz in macrophages (PMID: 275726050), this group's prior work identifying Tip5 region (PMID: 35112666) as well as prior human genetics work implicating CTSZ in clinical TB (PMID: 21354459)

We agree that the present manuscript is not the first to implicate CTSZ in TB susceptibility. However, we feel that previous work, including our prior identification of the *Tip5* region might be best described as evidence of significant genetic association, not in-depth *in vivo* evidence supporting the functionalities of CTSZ in this context. Beyond providing additional clarity on this topic, we think that there are several key insights of interest to a general audience.

1) Human granuloma tissue samples provide a novel view of the host-pathogen interface in patients with TB, underscoring CTSZ as a central player that had been shown to be significantly associated with TB susceptibility. In the current iteration of this manuscript, we provide further validation of the human granuloma staining results with single-cell and spatially resolved RNA-Seq data from several patient-derived tissue samples, revealing *CTSZ* overexpression within the macrophage layer surrounding *Mtb*-containing pulmonary granulomas. Taken together, these staining and spatial scRNA-Seq results provide a high-precision snapshot of the human pulmonary granuloma and the placement of CTSZ within this vital immune structure during *Mtb* infection.
2) For the first time *in vivo*, we reveal the importance of *Ctsz* (as opposed to the broad *Tip5* region) in determining TB clinical outcomes. This finding not only validates prior human genetic association and *ex vivo* macrophage studies and but also serves as a proof-of-concept for the use of genetic reference populations, like the Collaborative Cross (CC) and Diversity Outbred (DO) populations, as a tool to identify critical disease-modifying genomic loci that can be mechanistically interrogated to provide translational insights into genetic factors with meaningful impact in human cohorts. Importantly, to our knowledge, this is the first functional study to validate a gene identified through a CC or DO mouse population in TB research, beyond bioinformatic prioritization or QTL mapping. While several studies have used these populations to map susceptibility loci, few have advanced to functionally confirm the causal gene or experimentally dissect its role during infection, a challenging but necessary step to follow-up large-scale infection screens. Beyond simply validating this candidate gene in mice, our work additionally provides a mechanistic link between CTSZ deficiency, early inflammatory responses, and long-term TB disease outcomes, bridging the gap from mouse genetic models to human patients. Considering the promise of the CC and DO mammalian genome reference panels that were created as a community resource over 15 years ago, this work is broadly important to highlight the intrinsic value and potential for translational discoveries in such large-scale community genome resources.
3) Per the penultimate Discussion paragraph, the importance of CTSZ in precipitating adverse disease outcomes is not isolated to TB or even to infectious diseases, as CTSZ has been proposed as a putative oncogene in previous studies (PMID: 21966391). As recently as March 2025, CTSZ was highlighted as one of three genetic drivers of worse prognosis in patients with gliomas using an unsupervised machine learning approach, adding to the evidence of CTSZ significance to both the fields of neuroscience and oncology (PMID: 40129966).
4) This manuscript includes discussions of the impact of diverse genetic background and now sex effects on quantitative traits, a topic with broad but vital implications for a general biology audience.

Separately, we maintain that TB is of interest to a general audience given the extent of the world thought to be currently infected, the recent increases in global TB burden, and the emergence of extensively drug-resistant strains (PMID: 39631498). Moreover, the fact that the book “Everything is Tuberculosis” by John Green has been on the New York Times Nonfiction Bestseller list for the last several weeks highlights that interest in TB is elevated among general and non-scientific audiences.

*	Mechanism is somewhat unclear both for how Ctsz is acting, and what the mutation is that is altering its expression in mice. Are there likely SNPs near Ctsz that might be altering expression? Does CTSZ directly restrict Mtb in the BMDMs they generated, or is it acting indirectly in vivo through cytokine release or other means?

We thank Reviewer #3 for these queries. To address the first question, we note that QTL mapping in the original CC infection screen used genotype array data, not whole-genome sequencing. As noted in the manuscript, this QTL mapping study led to the identification of the *Tip5* QTL, and within this *Tip5* locus, genomic inheritance from the NOD/ShiLtJ (NOD) line was associated with TB susceptibility. Although SNP data from the infected CC cohort are not directly available, we have performed comparison of the 8 CC/DO founder genome reference sequences across the *Tip5* region, which allowed us to infer whether SNPs unique to the NOD haplotype (to the exclusion of the other 7 founders) could exist in the *Tip5* region in CC mice. At the time of this comparison, we identified unique 7 SNPs in murine *Ctsz* that were harbored by only NOD founder mice, including 2 SNPs within exons and one SNP in the 3’UTR (a region of significance for human *CTSZ* in TB susceptibility, PMID: 21368909). Given 7 potential SNPs in the CC lines with NOD-derived *Tip5*, we decided to move forward with studying *Ctsz* on a B6 background to limit the impact of surrounding genetic variation and CC genomic mosaicism on interpretation of the biological impact of complete *Ctsz* ablation.

For the second question, in a study previously cited by reviewer #3 (PMID: 27572605), knockdown of *CTSZ* in human THP-1 macrophages was associated with increased *Mtb* survival. Recent evidence suggests that *Mtb* may directly inhibit the activity of most cathepsins as a method to inhibit phagosome activity and increase intracellular survival (PMID: 36674655). Generally, we have not observed significant differences in CFU between BMDMs from *Ctsz^–/–^* and *Ctsz^+/+^* sibling mice at 24 hours post-infection in our *ex vivo* model, with differences being primarily observed in CXCL1 levels. During *in vivo* infection, we generally see elevated CXCL1 in *Ctsz^–/–^* mice, with significantly elevated levels of CXCL1 observable even in uninfected *Ctsz^–/–^* lung homogenate (see below). We suggest that CXCL1 dysregulation is a signature phenotype of *Ctsz^–/–^* mice and will be the subject of future mechanistic study.

Minor Issues

*	Fig 2C- Many of these changes are likely to be consequence of higher bacterial burden rather than CTSZ directly; unclear exactly what is being plotted on heatmap. Log2FC?

We thank Reviewer #3 for this query. The values in the plot are mean values of the traits in each genotype at each timepoint that have been scaled and centered to enable inter-trait comparison on a heatmap. Without these adjustments, values like 1000 pg/mL would not be comparable in heatmap format next to 3.50 log_10_ CFU. The aim is to visualize the change in the levels of each trait between genotypes and timepoints, not necessarily show the raw values of each trait.

Further, we agree that the purpose of the panel depicted in Figure 2C was simply to observe the inflammatory microenvironment of the lungs of *Ctsz^–/–^* vs B6 at each assayed stage of infection. We made efforts in the text not to attribute causality to any one result in the panel, although Reviewer #3 is correct that several of these results are likely associated with elevated burden in *Ctsz^–/–^* mice. However, authors note that even at week 3 post-infection, when lung burden was not statistically differentiable between B6 and *Ctsz^–/–^* mice (Fig 2A, p=0.7), we still see enhanced lung CXCL1 in *Ctsz^–/–^* mice (Fig 2F, p=4.7e-06), suggesting that enhanced burden is likely not the sole driver of this enhanced inflammatory response. Despite this divergence between burden and inflammation, we find from sPLS-DA analysis that CXCL1 and bacterial burden are both signatures of disease in *Ctsz^–/–^* mice.

To explore inflammation driven by genetic ablation of *Ctsz* and to minimize the confounding effects of bacterial burden, we leveraged an *ex vivo* macrophage model in which we applied identical infectious doses to identically prepared cells of sibling mice, one with and one without functional *Ctsz*. In the initial pilots for these BMDM experiments, we assayed post-infection CFU. Across these pilot infections, we never observed any differences in CFU per plate well between *Ctsz^+/+^* and *Ctsz^–/–^* siblings within the same MOI, suggesting that inflammation in this model cannot be driven by bacterial burden levels alone. Only after performing burden-controlled experiments, with the support of other published scRNA-Seq datasets, do we establish that *Ctsz* may be linked to CXCL1 levels in macrophages following infection.

*	Fig 2G- no statistics shown

We thank Reviewer #3 for this point. We have added details on hypothesis testing, which was performed via log-rank test, to the legends of Kaplan-Meier analyses in Figure 2 and Figure S2. Additionally, the p-values are now provided in each survival curve plot instead of in the main text and legend for Figure 2 and Figure S2, respectively.

*	Fig 3- these are relatively modest changes in cytokine levels

We thank Reviewer #3 for this observation. While differences in cytokine concentrations between *Ctsz^+/+^* and *Ctsz^–/–^* siblings may appear modest, it is well-established that even small shifts in cytokine levels can trigger substantial downstream effects on cellular recruitment, activation, and the overall immune landscape during TB infection (PMID: 2788309, 28739876). Studies in human TB patients, for example, have shown that even small increases in CXCL1 correlate with higher bacterial burdens in sputum smears and more severe disease features, including bilateral lung involvement and cavitation (PMID: 31796883), which underscores that even seemingly modest shifts in CXCL1 may have meaningful downstream effects on TB disease progression.

*	Fig 2 - looks at mouse CTSZ protein levels but not RNA, figure 4 looks at human CTSZ RNA levels but not protein. Would be helpful to link, perhaps by examining CTSZ RNA in mouse lungs or BMDMs alongside protein.

As with any translational study, it is challenging to directly match human clinical samples and readouts with pre-clinical *in vivo* results. Unfortunately, protein samples were not available from the human monocyte study, and samples for transcript quantification were not collected from the mouse or BMDM infection experiments. To strengthen the connection to human TB and bridge the gap between RNA and protein results in patients with TB, we now include newly generated single-cell, spatially resolved transcriptomic data and analysis from human TB granulomas. These results highlight elevated expression of *CTSZ* in the epithelioid macrophage layer surrounding human TB granulomas, consistent with CTSZ antibody staining results from human TB granulomas included in the initial submission. We include entirely new panels with these results and analyses (Figure 4E-H and Figure S4), which validate the positioning of CTSZ at the host-pathogen interface within human lungs infected with *Mtb*.

*	Fig 4D - these seem like fairly small changes but hard to tell on Log2 scale. Linear scale probably more appropriate with such small differences.

Even for significant SNPs, small effect sizes are a common feature of human GWAS results (PMID: 20562874). For consistency with changes suggested by Reviewer #1 and with other plots throughout the manuscript, we feel that it is appropriate to keep the log-transformation for Figure 4D.

*	Fig 4E probably would be useful to show some non-macrophage marker as a control (TCR? CD20?)

We thank Reviewer #3 for this recommendation. If the intention behind including a non-macrophage marker would be to highlight cells that are negative for CD68 (and CTSZ), we do include DAPI, a DNA stain that highlights cell nuclei, in this panel. In the Overlay figure on the far right, there are visible nuclei that lack any positive staining for CD68 or CTSZ, highlighting that CD68-negative cells (non-macrophages) are present in the imaged sample without the need for additional staining controls. Moreover, newly included scRNA-Seq data in Figure 4E-H and Figure S4 clearly illustrate that *CTSZ* is specifically and robustly expressed in human granuloma-associated macrophages, but largely not in other assayed cell types.
